# Effect of a chemical inhibitor of human phosphatidylethanolamine-binding protein 4 on radiosensitivity of rectal cancer cells

**DOI:** 10.1186/s12957-016-0977-3

**Published:** 2016-08-23

**Authors:** Jianming Qiu, Yong Tao, Guangen Yang, Kan Xu, A. Li Lin, Liuyu Li

**Affiliations:** 1 Department of Colorectal Surgery, The Third Hospital of Hangzhou, 38 West Lake Avenue, ShangChen District, Hangzhou, China; 2 Department of Pathology, The Third Hospital of Hangzhou, 38 West Lake Avenue, ShangChen District Hangzhou, China

**Keywords:** Human phosphatidylethanolamine-binding protein 4, Radiosensitivity, Rectal cancer

## Abstract

**Background:**

Human phosphatidylethanolamine-binding protein 4 (hPEBP4) is a well-established antiapoptosis molecule in recent years. It has also been demonstrated to be involved in the radioresistance of rectal cancer. The objective of this study was to determine whether IOI-42, a chemical inhibitor of hPEBP4, could sensitize rectal cancer cells.

**Methods:**

Rectal cancer cells were treated with IOI-42 alone or in combination with irradiation. Clonogenic survival assays and tumor volume growth analysis were used, respectively, to study the effect of IOI-42 in vitro and in vivo. Western blot was adopted to measure the activation of signal pathway.

**Results:**

Clonogenic survival assays showed that IOI-42, combined with irradiation, caused a significant decrease in colony formation compared with radiation alone, which was associated with the downregulation of Akt activation. And we also confirmed the effect of IOI-42 in nude mice transplanted with human rectal cancer subcutaneously.

**Conclusions:**

These data suggest that IOI-42 has a potential to enhance the radiosensitivity of rectal cancer cells, providing a rationale to further investigate the feasibility of combining of IOI-42 with radiation, keeping in mind that this may result in unexpected toxicities.

## Background

The phosphatidylethanolamine-binding protein (PEBP) family consists of a number of 21–23-kDa basic proteins, first identified in the bovine brain, with preferential in vitro affinity for phosphatidylethanolamine, a component of the cell membrane [1]. A number of functions have been suggested for the mammalian PEBP proteins, including lipid binding and inhibition of serine proteases [2]. Human phosphatidylethanolamine-binding protein 4 (hPEBP4) is a novel member of the PEBP family [3]. A recent study shows that hPEBP4 was an independent predictive biomarker for the response of rectal cancer to preoperative radiotherapy [4]. The radioresistance effect of hPEBP4 has been demonstrated to be associated with Akt activation [5]. At the very beginning, the study on hPEBP4 has focused on its antiapoptosis effect [4, 6–9]. A specific chemical inhibitor of hPEBP4 was also found through virtual screening, which showed significant antiapoptosis effect by targeting the conservative PE-binding domain of the molecule of hPEBP4 [10]. Thus, we speculated that this inhibitor named as IOI-42 may also enhance the radiosensitivity of rectal cancer. In this study, we evaluated the radiosensitizing effect of IOI-42 both in vitro and in vivo using the model of rectal cancer cells and nude mice transplanted with human rectal tumor, respectively.

## Methods

### Antibody and cells

Antibodies specific to phospho-Akt (Ser473) were from Cell Signaling Technology (Beverly, MA). LY-294002 was purchased from Sigma-Aldrich (Shanghai, China). All human rectal cancer cells including HRT18 and HT-29 were purchased from the American Type Culture Collection (Manassas, VA) and maintained in a DMEM medium (Gibco, USA) supplemented with 10 % (*v*/*v*) fetal calf serum, 4.5 g/l d-glucose, nonessential amino acids (100 mM each), 100 units/ml penicillin, 100 mg/ml streptomycin, and 2 mM glutamine at 37 °C in a 5 % CO_2_ atmosphere.

### Chemical inhibitor of hPEBP4

The structure of IOI-42 was identical as that shown in the original study [8]. A 10-mM stock solution was prepared in sterile 0.9 % sodium chloride and stored at 4 °C. In this study, IOI-42 was used at increasing concentrations from 1 to 10 μM.

### Clonogenic survival assay

Exponentially growing rectal cancer cells in monolayer culture were irradiated in 100-mm Petri dishes using a 250-kVp X-ray (0.61 Gy/min) by single radiation exposure. After exposure to ionizing irradiation, the cells were harvested for clonogenic survival analysis. Survival after radiation exposure was defined as the ability of the cells to maintain their clonogenic capacity and form colonies. Briefly, after exposure to radiation, cells were trypsinized, counted, and seeded for colony formation in 100-mm Petri dishes at 500–1000 cells/dish. After incubation intervals of 21 days, colonies were stained with crystal violet and counted manually. Colonies consisting of more than 50 cells were scored, and three replicate dishes were counted for each treatment.

Experiments were carried out at least three times for all data points. The experimental results were corrected for effects induced by the nonirradiated control.

### In vivo radiation studies

Male athymic nu/nu mice (4 weeks old) were purchased from the Experimental Animal Center of Shanghai and were raised using protocols that had been approved by the Institutional Animal Care and Use Committee. Forty-eight mice were randomly divided into four groups. The mice were injected subcutaneously with 2 × 10^6^ HRT-18 cells on the right foot pad. When tumors were approximately 0.5–0.8 cm in the longest diameter, the mice of four groups were subject to no treatment, irradiation alone, irradiation with IOI-42 intratumoral injection, or IOI-42 alone. Irradiation regimen was as the following: 2 Gy Cs-radiation source every 3 days for three times as described before [11]. Tumors were monitored and measured with the formula *V* = (*a* × *b*^2^)/2 every 3 days. Observation was closed once the average volume of tumor of any group reached 3.0 cm^3^. All mice were executed 33 days after radiation exposure, and tumor size-time curve as well as the apoptosis of tumor tissues was analyzed.

### Immunohistochemistry analysis of Akt activation and expression of DNA repair genes

Transplanted human rectal cancer tissues were obtained from the executed mice. The specimens were fixed with formalin, embedded in paraffin, and immunostained with specific antibody using avidin-biotin peroxidase complex method. Immunoreactivity was evaluated based on the percent of positive-stained cells.

### TUNEL assay

Transplanted tumors from mice were collected and fixed in 10 % formalin and embedded in paraffin after mice were excised. After deparaffinization, the tumor samples were stripped of protein by incubation with 20 mg/ml proteinase K (Sigma Chemical) for 15 min at room temperature. TUNEL staining was performed using an apoptosis in situ detection kit (TaKaRa) according to the manufacture’s protocols. The frequency of apoptosis was calculated as an apoptotic index, in which more than 200 positive cells were examined in five random fields of each group, and TUNEL-positive cells were counted to calculate the percent of positive cells as the apoptosis index in situ.

### Statistical analysis

Data are expressed as mean values ± SD. Statistical significance of differences between groups was tested by Student’s *t* test, one-way ANOVA, or chi-square test. A *p* value of less than 0.05 was considered significant.

## Results

### Effects of IOI-42 on clonogenic survival of rectal cancer cells after irradiation

**Fig. 1:**
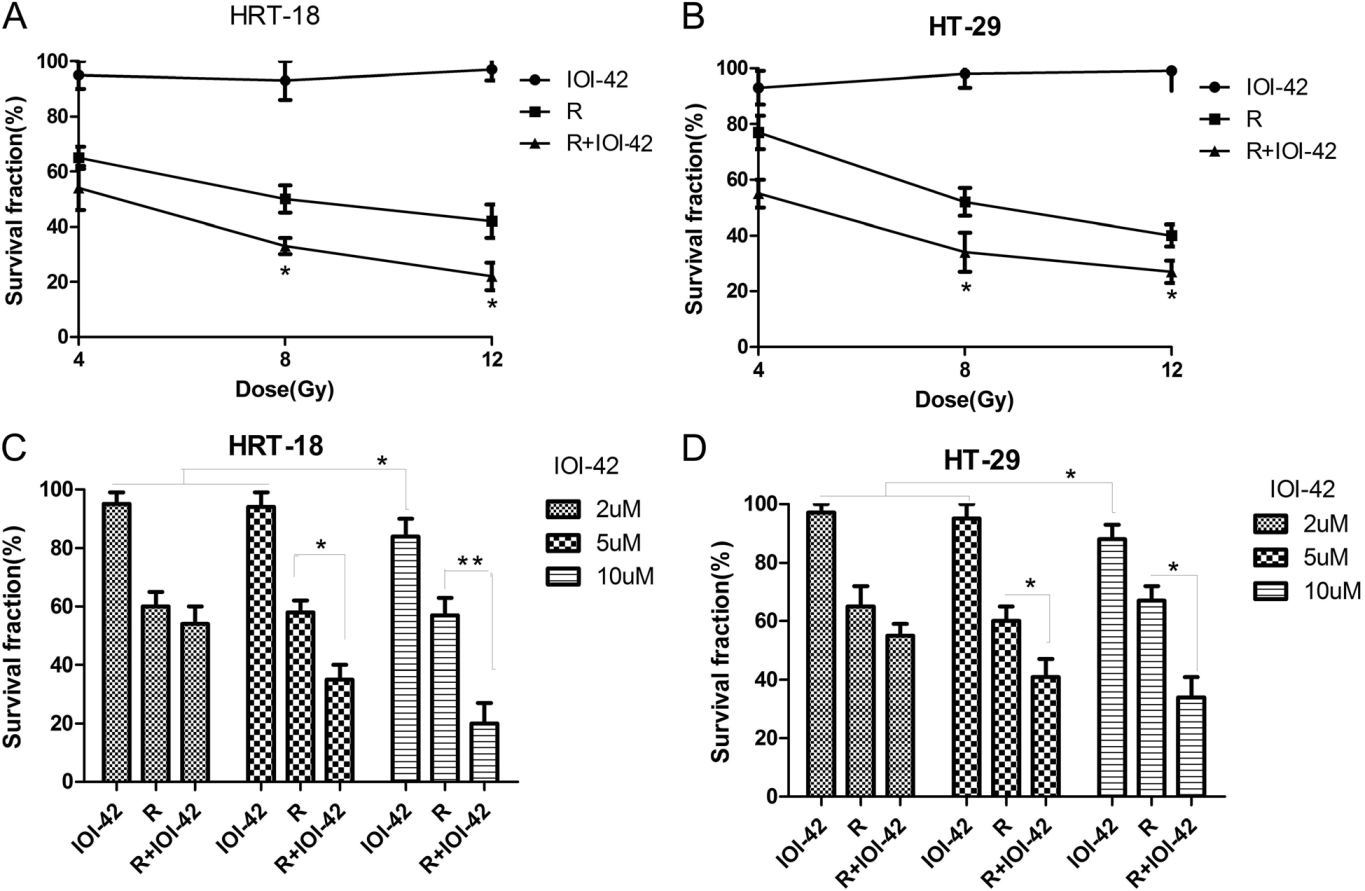
Effects of IOI-42 on clonogenic survival of rectal cancer cells: **a** HRT-18 cells. **b** HT-29 cells with irradiation doses of 4, 8, and 12 Gy, combined with (or not) 5 μm IOI-42. **c** HRT-18 cells. **d** HT-29 cells with different concentrations of IOI-42 combined with 8-Gy radiation

In order to evaluate the radiosensitizing effect of IOI-42 on rectal cancer in vitro, we examined the effect of IOI-42 on the clonogenic survival of two rectal cancer cell lines combined with irradiation. We found that IOI-42 itself did not influence the survival of both HRT-18 and HT-29 cells. But it significantly enhanced the killing of rectal cancer cells by irradiation (Fig. 1a, b). Then, we investigated the concentration dependence inhibition of colony formation of these cell lines for different concentrations of IOI-42. As the concentration of IOI-42 increases, the survival of rectal cancer cells decreased after irradiation, and the higher the IOI-42 concentration, the lower the survival of rectal cancer cells (*p* < 0.05). However, when increased to a concentration of 10 μM, IOI-42 itself significantly reduced the survival of rectal cancer cells (Fig. 1c, d). So in the following experiments, we chose to study the radiosensitizing effect of IOI-42 at the concentration of 5 μM.

### IOI-42-mediated radiosensitivity of rectal cancer cells was Akt dependent

**Fig. 2:**
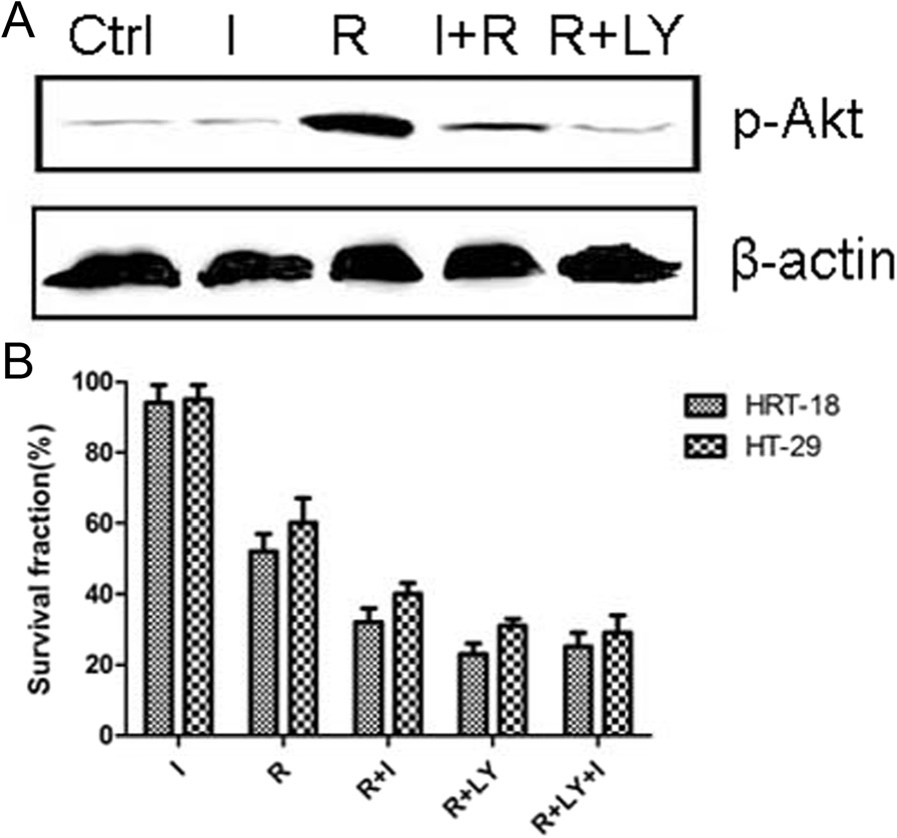
IOI-42-mediated radiosensitivity of rectal cancer cells was Akt dependent. **a** Effect of radiation, IOI-42, and LY-294002 on the activation of Akt. **b** LY-294002 abolish the effect of IOI-42 both in HRT-18 and HT-29 cells. (*I* is the short form for IOI-42, *R* is the short form for irradiation)

Akt activation has been demonstrated to be essential in the hPEBP4-mediated radioresistance of rectal cancer [5], so we explored the role of Akt in the effect of IOI-42. Firstly, we confirmed the activation of Akt after irradiation, its dramatic inhibition by IOI-42, and its almost complete depression by pre-incubation with 20 μM LY-294002 as well (Fig. 2a). Then, we examined the role of Akt activation in the radiosensitizing effect of IOI-42. We found that the effect of IOI-42 was totally abolished after LY-294002 inhibited activation of Akt for both HRT-18 and HT-29 cells (Fig. 2b).

### IOI-42 promoted the sensitivity of rectal cancers to irradiation in vivo

**Fig. 3:**
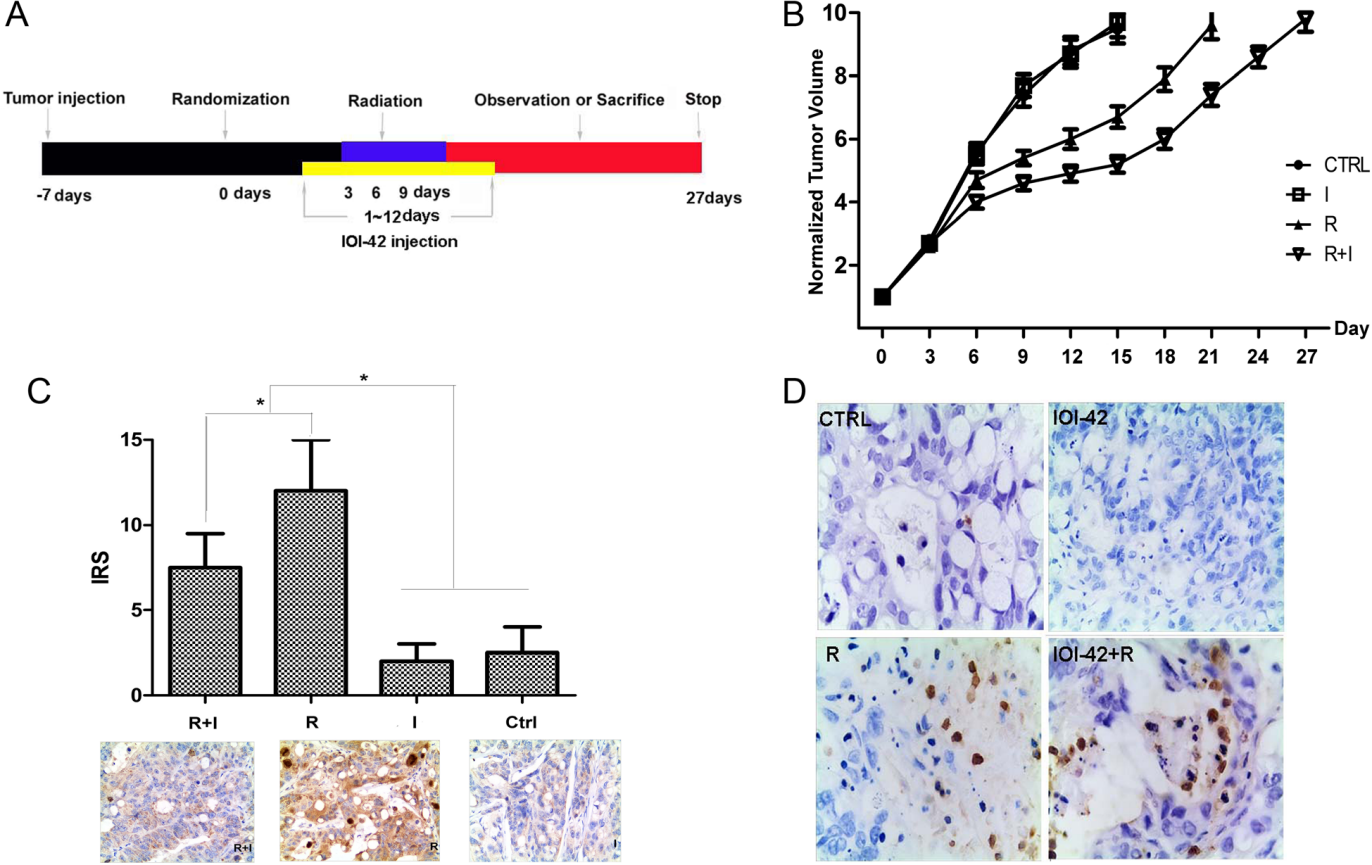
IOI-42 promoted the sensitivity of rectal cancers to irradiation in vivo. **a** Flow chart for the in vivo radiation experiment. **b** Growth curve of transplanted rectal cancer volume for different treatments. **c** Immunoreactive score for Akt activation in vivo after different treatments (below is the representative pictures of activated Akt staining of radiation plus IOI-42, radiation alone, and IOI-42 alone). **d** Representative pictures of TUNEL assay for different treatments. (*I* is the short form for IOI-42, *R* is the short form for irradiation, *IRS* is the short form for immunoreactive score)

To determine whether IOI-42 can also promote the radiosensitivity of colorectal cancer in vivo, we examined the effect of radiation alone, IOI-42 alone, or in combination on the growth of subcutaneous HT-29 xenograft rectal tumors in nude mice (Fig. 3a). We found that from the 12th day, the tumor volume in the combined treatment group was significantly smaller than that in the radiation only group (*p* < 0.05). And no growth delay was observed in the IOI-42 only group compared with control (Fig. 3b). We also examined Akt activation of the tumor tissue with immunohistochemistry, which shows that IOI-42 significantly compromised irradiation-induced Akt activation (Fig. 3c). The effect of IOI-42 on the rectal tumor growth after irradiation was also confirmed by apoptosis stain in situ (Fig. 3d); the apoptosis index of the combined group is significantly higher than that of the group of radiation alone (1.5 vs 0.85, *p* < 0.05).

## Discussion

As the first chemical inhibitor of hPEBP4, IOI-42 has been demonstrated to be able to block the conservative PE-binding domain of hPEBP4 and reverse the signal pathway affected by hPEBP4 overexpression [10]. In the present study, we proved that IOI-42 could enhance the radiosensitivity of rectal cancer cells both in vitro and in vivo through inhibiting hPEBP4-induced Akt activation after irradiation.

Since hPEBP4 has been shown to be overexpressed in breast, prostate, and ovarian cancers [3, 6–9], our study suggested that IOI-42 might also be a potential radiosensitizing agent for all the involved human cancers. There have been seldom breakthrough in the development of radiosensitizing agents in recent years. To speed up the development of radiosensitizing agents, taking advantage of the differentially expressed gene profile of cancer rather than just focusing on some classical death signal pathway might be essential [12–15].

Consistent with previous study with siRNA to silence hPEBP4 [3], our study confirmed that inhibition of Akt activation is pivotal in the radiosensitizing effect of IOI-42. The upregulation of Akt activation by hPEBP4 was believed to be reactive oxygen species (ROS)-dependent, though we did not know the exact signal event downward of ROS, through which hPEBP4 activated Akt to promote the radioresistance of rectal cancer [5, 7]. Neither we know the final effect molecule after Akt activation. One thing is for sure that targeting the conservative PE-binding domain of the molecule of hPEBP4 is essential for IOI-42 in playing its radiosensitizing effect. To address that problem, we actually compared the expression of some nucleotide repair genes between irradiation alone and combination of irradiation with IOI-42 in this study but found no significant difference for nucleotide repair genes like FANCG, ERCC1, PMS1/2, BRCA1/2, LIG4, and TP53 [16–20]. So the detailed mechanism of hPEBP4-induced radioresistance needs further exploration, which will promote the development of more chemical inhibitors of hPEBP4 and also the potential application of multi-targeting chemicals with stronger radiosensitizing effect. Being a preliminary study of IOI-42 as a radiosensitizing agent for rectal cancer, we did not examine the side effect of IOI-42. But we did observe anorexia in some mice subject to IOI-42 when compared with control. It requires systematic research to clarify its potential toxic effect if IOI-42 turned out to be a promising agent to enhance radiosensitivity.

## Conclusions

In summary, this is the very first report on a chemical inhibitor of hPEBP4 in promoting the radiosensitivity of rectal cancer, suggesting IOI-42 may be a potential radiation-sensitive agent in the clinical treatment of cancers. Further work is necessary to demonstrate its efficacy on other human cancers and also to evaluate its possible side effect.

## Abbreviation

hPEBP4: Human phosphatidylethanolamine-binding protein 4
